# Identification of *ACBD3* as a new molecular biomarker in pan-cancers through bioinformatic analysis: a preclinical study

**DOI:** 10.1186/s40001-023-01576-8

**Published:** 2023-12-14

**Authors:** Xinyue Ma, Shu Huang, Huiqin Shi, Rui Luo, Bei Luo, Zhenju Tan, Lei Shi, Wei Zhang, Weixing Yang, Xiaolin Zhong, Muhan Lü, Xia Chen, Xiaowei Tang

**Affiliations:** 1 Department of Gastroenterology, The Affiliated Hospital of Southwest Medical University, Street Taiping No. 25, Region Jiangyang, Luzhou, 646099 Sichuan China; 2 Nuclear Medicine and Molecular Imaging Key Laboratory of Sichuan Province, Luzhou, China; 3 Department of Gastroenterology, Lianshui County People’s Hospital, Huaian, China; 4 Department of Gastroenterology, Lianshui People’s Hospital of Kangda College Affiliated to Nanjing Medical University, Huaian, China; 5 Department of Gastroenterology, Clinical Medical College and the First Affiliated Hospital of Chengdu Medical College, Street Baoguang No.278, Region Xindu, Chengdu, 610500 Sichuan China

**Keywords:** *ACBD3*, *GCP60*, *GOCAP1*, Pan-cancer, Bioinformatic analysis, Biomarker

## Abstract

**Background:**

Acyl-CoA-binding domain-containing 3 (*ACBD3*) is a multifunctional protein, that plays essential roles in cellular signaling and membrane domain organization. Although the precise roles of *ACBD3* in various cancers remain unclear. Thus, we aimed to determine the diverse roles of *ACBD3* in pan-cancers.

**Methods:**

Relevant clinical and RNA-sequencing data for normal tissues and 33 tumors from The Cancer Genome Atlas (TCGA) database, the Human Protein Atlas, and other databases were applied to investigate *ACBD3* expression in various cancers. *ACBD3*-binding and *ACBD3*-related target genes were obtained from the STRING and GEPIA2 databases. The possible functions of *ACBD3*-binding genes were explored using Gene Ontology (GO) and Kyoto Encyclopedia of Genes and Genomes (KEGG) enrichment analyses. We also applied the diagnostic value and survival prognosis analysis of *ACBD3* in pan-cancers using R language. The mutational features of *ACBD3* in various TCGA cancers were obtained from the cBioPortal database.

**Results:**

When compared with normal tissues, *ACBD3* expression was statistically upregulated in eleven cancers and downregulated in three cancers. *ACBD3* expression was remarkably different among various pathological stages of tumors, immune and molecular subtypes of cancers, cancer phosphorylation levels, and immune cell infiltration. The survival of four tumors was correlated with the expression level of *ACBD3*, including pancreatic adenocarcinoma, adrenocortical carcinoma, sarcoma, and glioma. The high accuracy in diagnosing multiple tumors and its correlation with prognosis indicated that *ACBD3* may be a potential biomarker of pan-cancers.

**Conclusion:**

According to our pan-cancer analysis, *ACBD3* may serve as a remarkable prognostic and diagnostic biomarker of pan-cancers as well as contribute to tumor development. *ACBD3* may also provide new directions for cancer treatment targets in the future.

**Supplementary Information:**

The online version contains supplementary material available at 10.1186/s40001-023-01576-8.

## Background

Most cancers are diagnosed at an advanced stage with a low chance of cure. The recent research on cancer biomarkers has provided a basis for cancer diagnosis and prognostic assessment, offering new opportunities for the survival of cancer patients [1–3]. Therefore, pan-cancer analyses of any possible genes are necessary to further investigate their molecular mechanisms and to determine their correlation with cancer prognosis.

Acyl-CoA binding domain-containing proteins (*ACBDs*) are made up of seven *ACBD* proteins that are essential for the transport and stabilization of acyl-CoA, cellular lipid metabolism, and organelle contact sites, and thus plays an important role in cell metabolism [4]. *ACBDs* have recently been considered key regulators in the development and progression of some cancers, including breast cancer, hepatocellular carcinoma, etc. [5–7] Acyl-CoA-binding domain-containing 3 (*ACBD3*) is a part of the *ACBD* family and a 528 amino acid residue protein [8]. Its vital biological feature is its interaction with different proteins [1]. *ACBD3* is also known as Golgi complex-associated protein 1 (*GOCAP1*), Golgi phosphoprotein 1(*GOLPH1*), Golgi complex-associated protein of 60 kDa(*GCP60*), and cAMP-dependent protein kinase and peripheral-type benzodiazepine receptor-associated protein 7(*PAP7*) [6, 9]. The various designations for *ACBD3* reflect its most prominent biological properties, such as transport and transfer of lipids, maintenance of Golgi integrity, regulation of steroidogenesis, and replication of the picornavirus family [4, 6, 10]. *ACBD3* is also a crucial player in membrane domain organization and cellular signaling [3]. Existing studies have shown that *ACBD3* mediates the malignant process of breast cancer by regulating the intracellular β-84 catenin signaling pathway [11]. *ACBD3* also affects the replication of gastric cancer cells in an AKT-dependent manner [12]. In addition, *ACBD3* may be involved in the progress of gefitinib on lung cancer cells [13].

Previous researches have demonstrated that *ACBD3* played a significant role in the development and treatment of different cancers [11–13]. No studies have explored the association between *ACBD3* and pan-cancer development until now. The aim of this study was to further explore whether *ACBD3* could serve as a new pan-cancer biomarker and to further determine its molecular mechanism and prognostic relevance. This research was conducted to investigate *ACBD3* expression among pan-cancers, investigate the prognostic and diagnostic value of *ACBD3* among various tumors, and determine the correlation between *ACBD3* mutation characteristics and pan-cancer prognosis.

## Methods

### Gene expression analysis

We used the “TISSUE” module of Human Protein Atlas (THPA) database (https://www.proteinatlas.org) in December 2022, which works to provide information on the tissue and cellular distribution of all 24,000 human proteins, to investigate *ACBD3* expression from the normal tissue atlas and tumor cell lines. And then explored *ACBD3* expression in cells using the “SUBCELL” module of THPA database. We downloaded the relevant clinical and RNA-sequencing (RNA-seq) data for normal tissues and 33 tumors from The Cancer Genome Atlas (TCGA) database on 23 February 2023 [14]. R software (version 4.2.1) and ggplot2 package (version 3.3.6) were performed to generate statistical analysis and visualizations, respectively. The Wilcoxon rank-sum test and T test were performed to compare the differences in *ACBD3* expression levels between cancer and normal tissues, and between cancer and paracancerous tissues. The Cancer Cell Line Encyclopedia (CCLE) database was used to verify gene expression in pan-cancers on 14 May 2023 [15]. Immunohistochemistry images of different cancers and normal tissues were obtained from the “TISSUE” and “PATHOLOGY” module of THPA database [16]. The data on the relationship between *ACBD3* expression and immune infiltrates among various tumors were obtained from the “Immune-Gene” module of TIMER2.0 database in December 2022, which used six state-of-the-art algorithms to provide more reliable estimates of immune infiltration levels for tumor profiles [17].

The *ACBD3* expression among various tumor pathological stages was visualized using the “Stage Plot” module of the gene expression profiling interactive analysis (GEPIA2) database (Used on 28 February 2023). *ACBD3* phosphoprotein levels in normal tissues and different cancers were obtained by searching for “PhosphoProtein” module of *ACBD3* from the Clinical Proteomic Tumor Analysis Consortium (CPTAC) database in March 2023 [18]. Furthermore, we entered *ACBD3* in the “Gene Symbol” module of the TISIDB (an integrated repository portal for tumor-immune system interactions) database [19], a website for analyzing interactions between tumors and the immune system, to observe the correlation between *ACBD3* expression and various molecular and immune subtypes among different cancers.

### *ACBD3*-related gene enrichment analyses

We obtained 50 available experimentally determined *ACBD3*-binding proteins from the Search Tool for the Retrieval of Interacting Genes/Proteins (STRING) database in February 2023 by searching *ACBD3* and Homo sapiens organism. We regulated the parameters as follows: “full STRING network”, “evidence”, “Experiments, Textmining, Databases”, “medium confidence (0.400)”, and “no more than 50 interactors” in the 1st shell. Subsequently, the protein − protein interaction (PPI) network was visualized using Cytoscape (version 3.9.1). Next, we retrieved the top 100 *ACBD3*-related target genes from the “Similar Gene Detection” module of the GEPIA2 database. A Venn diagram was constructed to compare *ACBD3*-binding and *ACBD3*-related target genes. Moreover, the possible functions of *ACBD3*-binding genes were explored using Gene Ontology (GO) and Kyoto Encyclopedia of Genes and Genomes (KEGG) enrichment analyses by the R package “ClusterProfiler” (version 4.4.4).

### Diagnostic value and survival prognosis analysis

We investigated the diagnostic and prognostic values of *ACBD3* using relevant pan-cancer clinical data from TCGA database. The diagnostic performance of *ACBD3* in pan-cancers was assessed using the receiver-operating characteristic (ROC) curve and the area under the ROC curve (AUC) applied by the “pROC” package (version 1.18.0). The included AUC values ranged from 0.7 to 1. As a measure of diagnostic accuracy, when the value of the AUC is closer to 1, the diagnostic value is higher. Furthermore, we constructed Kaplan–Meier (K-M) plots using the “survival” and “survminer” package (version 3.3.1) to demonstrate the relationship between *ACBD3* expression level and the prognosis [OS (overall survival), DSS (disease-specific survival), and PFI (progress-free interval)] of various tumors [20], the Gene Expression Omnibus (GEO) database was used to verify this relationship [21].

### Genetic alteration and DNA methylation analysis

The cBioPortal website was used to investigate genetic alterations in *ACBD3* on February 2023. Next, we searched out the mutation type, the alteration frequency, and the copy number alteration (CNA) of all TCGA tumors from the “Cancer Types Summary” module. Furthermore, we obtained the three-dimensional (3D) diagram of *ACBD3* structure by using the “Mutations” module. In addition, we also accessed the correlation between *ACBD3* alternation and clinical outcomes in different tumors by performing the “Comparison/Survival” module. “Meth-Exp correlation” module of DNA Methylation Interactive Visualization Database (DNMIVD) (http://www.unimd.org/dnmivd/) was used to assess the relationship between promoter methylation and *ACBD3* expression, and “MethSurv” (https://biit.cs.ut.ee/methsurv) was used to explore the effect of DNA methylation on tumor prognosis and the relationship between *ACBD3* expression and methylation levels [22, 23].

## Results

### *ACBD3* expression in pan-cancers

These results suggested that *ACBD3* was moderately expressed in the majority of normal tissues. *ACBD3* was highly expressed in the cerebral cortex, hippocampus, duodenum, small intestine, colon, gallbladder, pancreas, prostate, placenta, appendix, and bone marrow. *ACBD3* was expressed at low levels in the oral mucosa, liver, ovary, soft tissue, and adipose tissue (Additional file 1: Fig. S1A). The expression of *ACBD3* ranked high in breast cancer, kidney cancer, and myeloma tumor cell lines (Additional file 1: Fig. S1B). Intracellular *ACBD3* was mainly distributed in the Golgi apparatus (Additional file 1: Fig. S1C, D).

**Fig. 1:**
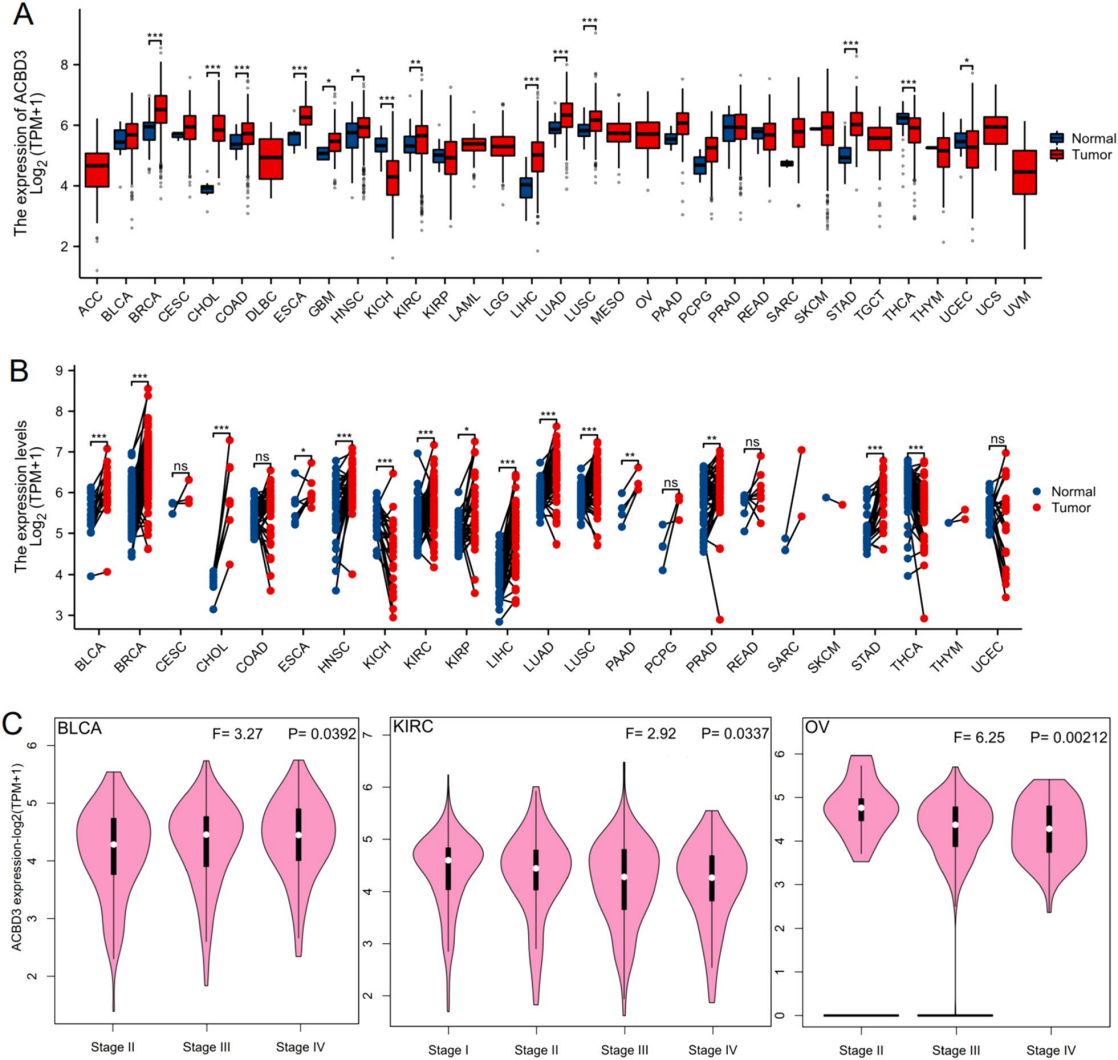
The expression level of *ACBD3* in normal tissues and different TCGA tumors. (*p < 0.05, **p < 0.01, ***p < 0.001). adrenocortical cancer (ACC), bladder cancer (BLCA), breast cancer (BRCA), cervical cancer (CESC), bile duct cancer (CHOL), colon cancer (COAD), large B-cell lymphoma (DLBC), esophageal cancer (ESCA), Glioblastoma (GBM), head and neck cancer (HNSC), kidney chromophobe (KICH), kidney clear cell carcinoma (KIRC), kidney papillary cell carcinoma (KIRP), acute myeloid leukemia (LAML), lower grade glioma (LGG), liver hepatocellular carcinoma (LIHC), lung adenocarcinoma (LUAD), lung squamous cell carcinoma (LUSC), mesothelioma (MESO), ovarian cancer (OV), pancreatic cancer (PAAD), pheochromocytoma & paraganglioma (PCPG), prostate cancer (PRAD), rectal cancer (READ), sarcoma (SARC), melanoma (SKCM), stomach cancer (STAD), testicular cancer (TGCT), thyroid cancer (THCA), thymoma (THYM), endometrioid cancer (UCEC), uterine carcinosarcoma (UCS), ocular melanomas (UVM). **A** *ACBD3* expression was statistically upregulated in eleven cancer types as compared to normal tissues, including BRCA, CHOL, COAD, ESCA, GBM, HNSC, KIRC, LIHC, LUAD, LUSC, STAD, and downregulated in KICH, THCA, and UCEC; **B** In comparison with the adjacent normal tissues, *ACBD3* expression was statistically upregulated in thirteen cancers, including STAD, PRAD, PAAD, LUSC, LUAD, LIHC, KIRP, KIRC, HNSC, ESCA, CHOL, BRCA, and BLCA, and downregulated in KICH and THCA; **C** The expression level of *ACBD3* in various pathological stages of BLCA, KIRC, and OV. *ACBD3* expressed the highest in stage III of BLCA, stage I of KIRC, and stage II of OV

**Fig. 2:**
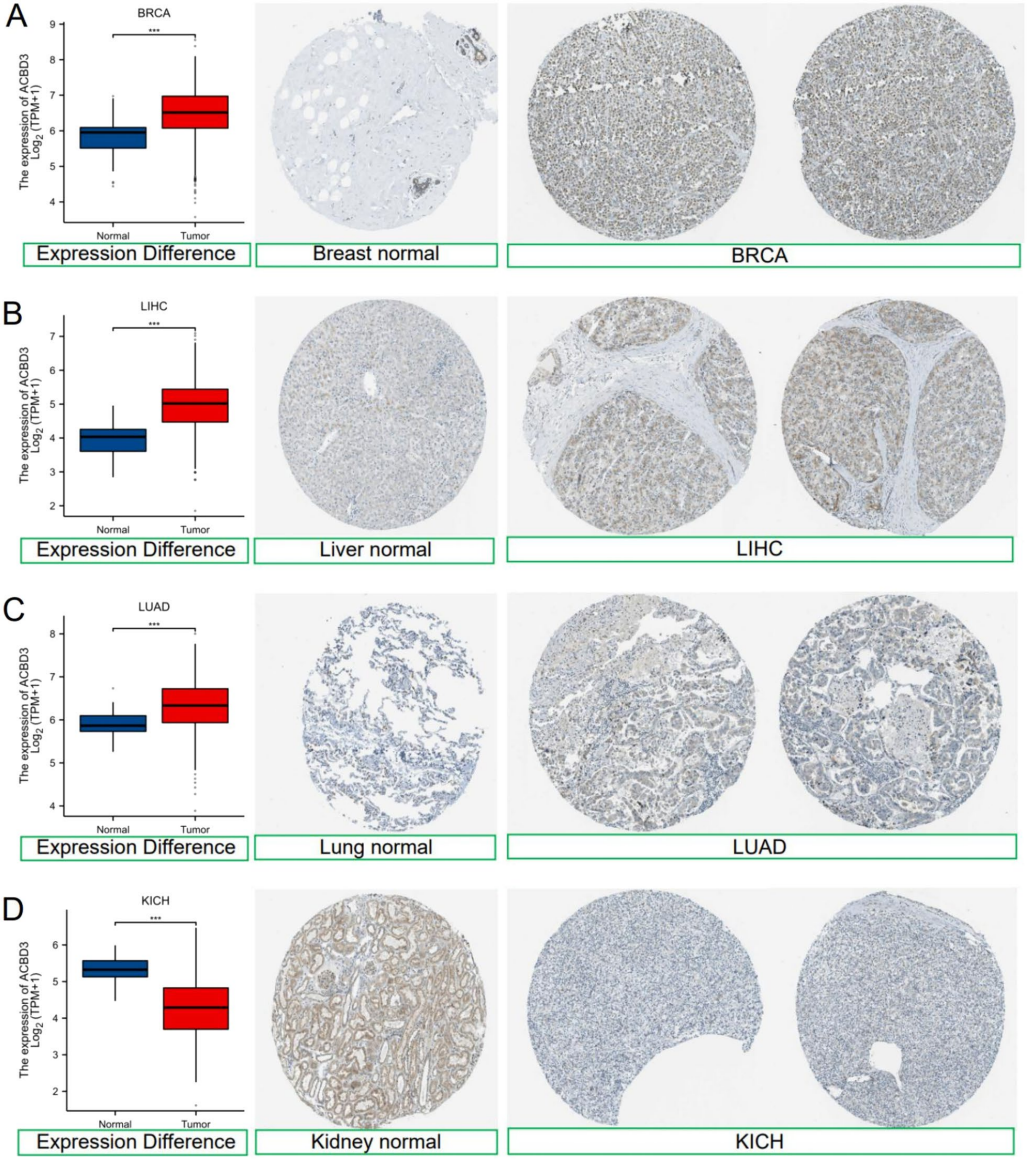
The expression level of *ACBD3* in different tumors and normal tissues (*p < 0.05, **p < 0.01, ***p < 0.001) and corresponding immunohistochemistry images. **A** *ACBD3* expression was statistically upregulated in breast invasive carcinoma (BRCA), **B** *ACBD3* expression was statistically upregulated in liver hepatocellular carcinoma (LIHC), **C** *ACBD3* expression was statistically upregulated in lung adenocarcinoma (LUAD), **D** *ACBD3* expression was statistically downregulated in kidney chromophobe (KICH)

Furthermore, we found that *ACBD3* expression was statistically upregulated in eleven cancer types as compared to normal tissues, including stomach adenocarcinoma (STAD), lung squamous cell carcinoma (LUSC), lung adenocarcinoma (LUAD), liver hepatocellular carcinoma (LIHC), kidney renal clear cell carcinoma (KIRC), head and neck squamous cell carcinoma (HNSC), glioblastoma multiforme (GBM), esophageal carcinoma (ESCA), colon adenocarcinoma (COAD), cholangiocarcinoma (CHOL), and breast invasive carcinoma (BRCA), and downregulated in uterine corpus endometrial carcinoma (UCEC), kidney chromophobe (KICH), and thyroid carcinoma (THCA) (Fig. 1A). The CCLE database revealed that *ACBD3* was highly expressed in skin cutaneous melanoma (SKCM), BRCA, GBM, KIRC, brain lower grade glioma (LGG), and THCA (Additional file 1: Fig. S1E). In comparison with the adjacent normal tissues, *ACBD3* expression was statistically upregulated in thirteen cancers, including STAD, prostate adenocarcinoma (PRAD), pancreatic adenocarcinoma (PAAD), LUSC, LUAD, LIHC, kidney renal papillary cell carcinoma (KIRP), KIRC, HNSC, ESCA, CHOL, BRCA, and bladder urothelial carcinoma (BLCA), and downregulated in KICH and THCA (Fig. 1B). We also constructed a violin diagram of the relationship between various pathological stages of tumors and *ACBD3* expression (Fig. 1C). Immunohistochemistry images of normal breast tissue, liver tissue, lung tissue, kidney tissue, BRCA, LIHC, LUAD, and KICH were displayed (Fig. 2).

We assumed that the different expression levels of *ACBD3* might affect the immune infiltration of various tumors. Therefore, we explored the correlation between *ACBD3* expression and immune infiltration in different cancers using the TIMER2.0 database. Remarkably, the expression of *ACBD3* was actively correlated with cancer-associated fibroblast (CAF) infiltration in HNSC (Additional file 1: Fig. S2A) and positively related to neutrophil infiltration in BLCA, COAD, and THCA (Additional file 1: Fig. S2B). *ACBD3* expression also positively related to endothelial cell infiltration in COAD, HNSC, and KIRC (Additional file 1: Fig. S2C).

**Fig. 3:**
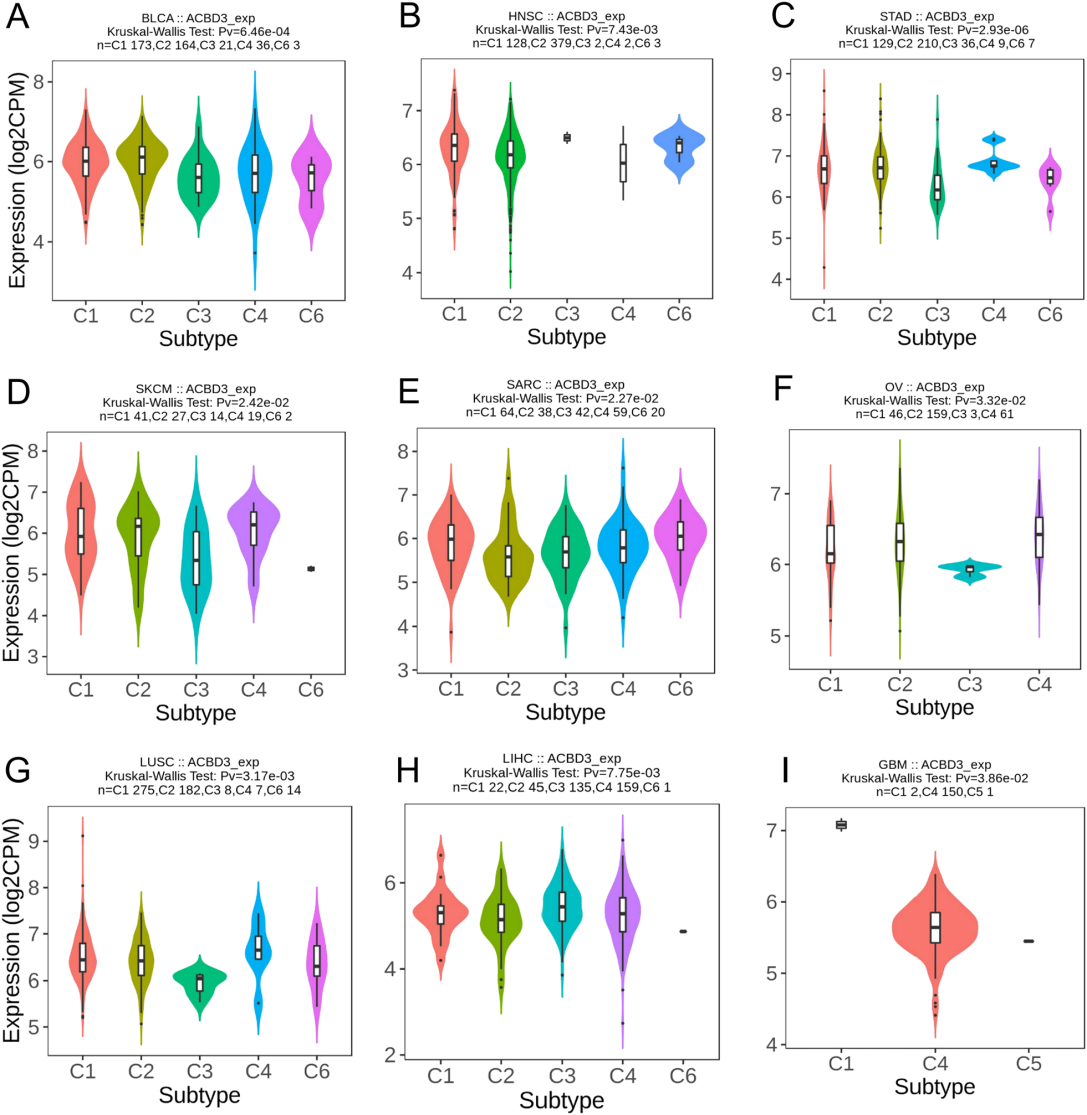
*ACBD3* expression in different immune subtypes of various TCGA cancers. Wound healing (C1), IFN-gamma dominant (C2), inflammatory (C3), lymphocyte depleted (C4), immunologically quiet (C5), TGF-b dominant (C6). **A** bladder urothelial carcinoma (BLCA), **B** head and neck squamous cell carcinoma (HNSC), **C** stomach adenocarcinoma (STAD), **D** skin cutaneous melanoma (SKCM), **E** sarcoma (SARC), **F** ovarian serous cystadenocarcinoma (OV), **G** lung squamous cell carcinoma (LUSC), **H** liver hepatocellular carcinoma (LIHC), **I** glioblastoma multiforme (GBM). For the immune subtype of C1, *ACBD3* expressed high in BLCA, SKCM, SARC, and LUSC. For the immune subtype of C2, *ACBD3* expressed high in HNSC, STAD, and OV. For the immune subtype of C4, *ACBD3* expressed high in LIHC, and GBM

Figure 3 showed that *ACBD3* expression was statistically different among the immune subtypes of nine tumors, including BLCA (Fig. 3A), HNSC (Fig. 3B), STAD (Fig. 3C), SKCM (Fig. 3D), sarcoma (SARC) (Fig. 3E), ovarian serous cystadenocarcinoma (OV) (Fig. 3F), LUSC (Fig. 3G), LIHC (Fig. 3H), and GBM (Fig. 3I). Moreover, for immune subtype C1 (wound healing), *ACBD3* expressed high in BLCA, SKCM, SARC, and LUSC. For immune subtype C2 (IFN-gamma dominant), *ACBD3* expressed high in HNSC, STAD, and OV. For immune subtype C4 (lymphocyte depleted), *ACBD3* expressed high in LIHC, and GBM.

**Fig. 4:**
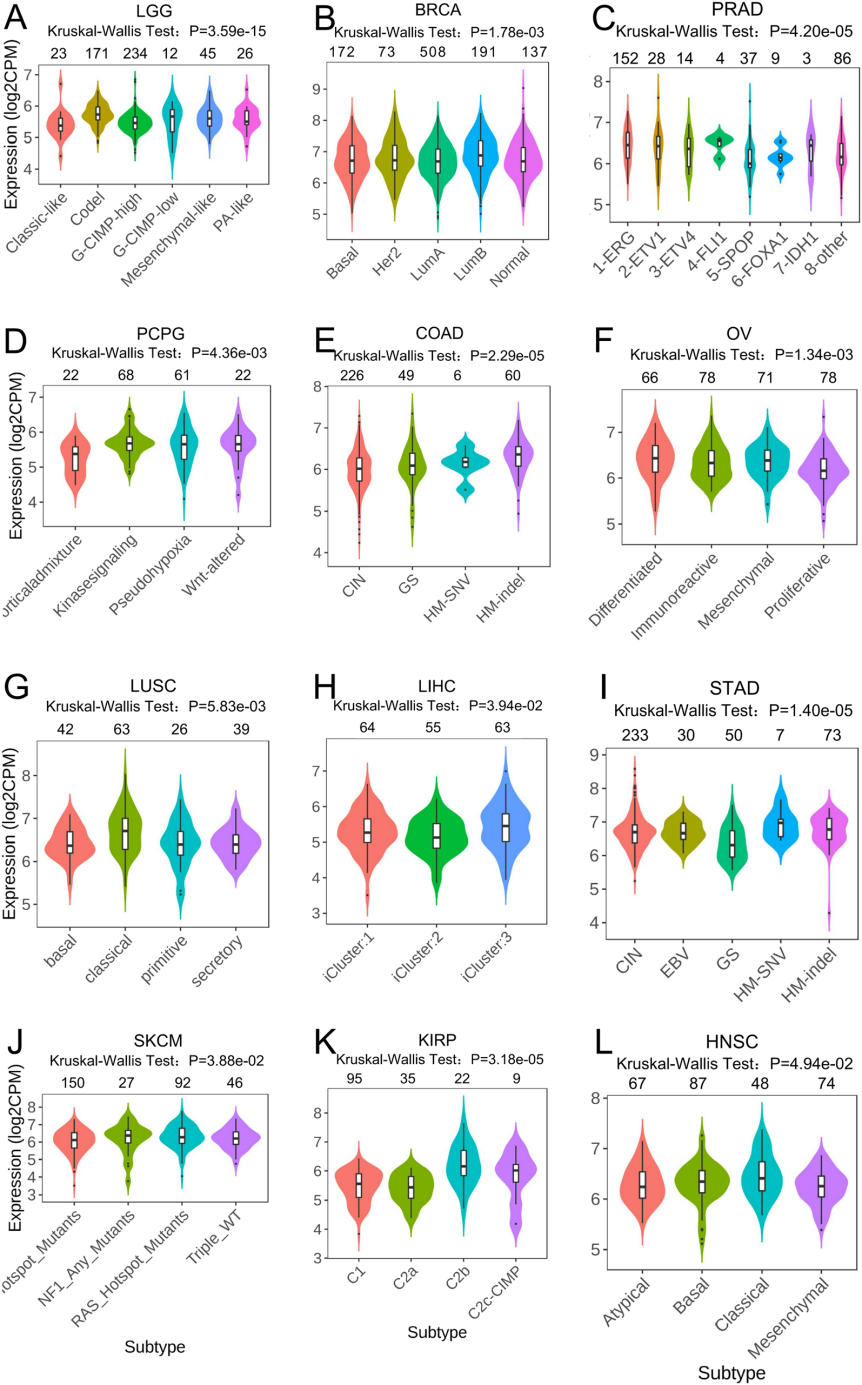
*ACBD3* expression in different molecular subtypes of various TCGA cancers. brain lower grade glioma (LGG), breast invasive carcinoma (BRCA), prostate adenocarcinoma (PRAD), pheochromocytoma and paraganglioma (PCPG), colon adenocarcinoma (COAD), ovarian serous cystadenocarcinoma (OV), lung squamous cell carcinoma (LUSC), liver hepatocellular carcinoma (LIHC), stomach adenocarcinoma (STAD), skin cutaneous melanoma (SKCM), kidney renal papillary cell carcinoma (KIRP), head and neck squamous cell carcinoma (HNSC). *ACBD3* expressed the highest in the molecular subtype of **A** G-CIMP-high in LGG, **B** LunA in BRCA, **C** 1-ERG in PRAD, **D** Kinasesignaling in PCPG, **E** CIN in COAD, **F** Immunoreactive and Proliferative in OV, **G** classical in LUSC, **H** iCluster:1 in LIHC, **I** CIN in STAD, **J** BRAF_Hotspot_Mutants in SKCM, **K** C1in KIRP, and **L** Basal in HNSC

Furthermore, we identified twelve tumors with molecular subtypes associated with *ACBD3* expression. Among the molecular subtypes of G-CIMP-high in LGG, *ACBD3* showed the highest expression (Fig. 4A). Among the molecular subtypes of LunA in BRCA, *ACBD3* showed the highest expression (Fig. 4B). Among the molecular subtypes of 1-ERG in PRAD, *ACBD3* showed the highest expression (Fig. 4C). Among the molecular subtypes of Kinasesignaling in pheochromocytoma & paraganglioma (PCPG), *ACBD3* showed the highest expression (Fig. 4D). Among the molecular subtype of CIN in COAD, *ACBD3* showed the highest expression (Fig. 4E). Among the molecular subtypes of Immunoreactive and Proliferative in OV, *ACBD3* showed the highest expression (Fig. 4F). Among the molecular subtypes of classical in LUSC, *ACBD3* showed the highest expression (Fig. 4G). Among the molecular subtypes of iCluster:1 in LIHC, *ACBD3* showed the highest expression (Fig. 4H). Among the molecular subtypes of CIN in STAD, *ACBD3* showed the highest expression (Fig. 4I). Among the molecular subtypes of BRAF_Hotspot_Mutants in SKCM, *ACBD3* showed the highest expression (Fig. 4J). Among the molecular subtypes of C1in KIRP, *ACBD3* showed the highest expression (Fig. 4K). Among the molecular subtypes of Basal in HNSC, *ACBD3* showed the highest expression (Fig. 4L).

### *ACBD3*-related gene enrichment analyses

**Fig. 5:**
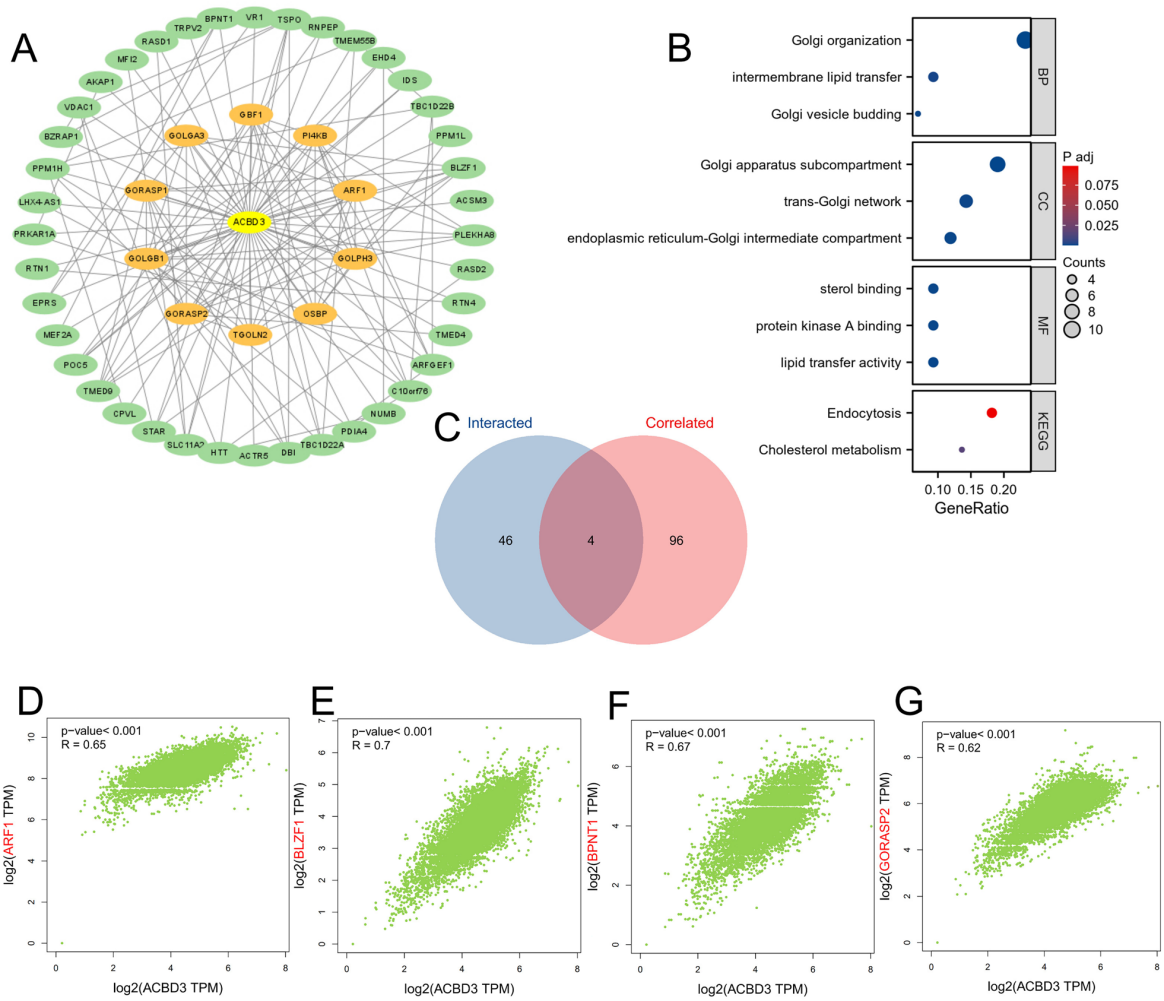
The correlated and interacted genes of *ACBD3*. **A** 50 targeted binding proteins of *ACBD3* involved in the protein–protein interaction network; **B** the Gene Ontology and Kyoto Encyclopedia of Genes and Genomes (KEGG) enrichment analyses of the 50 interacted genes of *ACBD3*, including biological process (BP), cellular component (CC), molecular function (MF), and KEGG; **C** The Venn diagram of the correlated and interacted genes of *ACBD3*; Correlation analysis between four intersection genes and *ACBD3*, including **D** *ARF1* (*R* = 0.65), **E** *BLZF1* (*R* = 0.70), **F** *BPNT1* (*R* = 0.67), and **G** *GORASP2* (*R* = 0.62)

We identified 50 *ACBD3*-binding proteins from the STRING database (Fig. 5A). Next, GO|KEGG enrichment analysis was conducted on the 50 proteins, revealing that the biological processes (BP) of the 50 proteins included Golgi organization, intermembrane lipid transfer, and Golgi vesicle budding. The cellular components (CC) of the 50 proteins were mainly involved in the Golgi apparatus subcompartment, endoplasmic reticulum-Golgi intermediate compartment, and trans-Golgi network. The molecular function (MF) of 50 proteins were primarily enriched in sterol binding, protein kinase A binding, and lipid transfer activity. The KEGG pathway enrichment of the 50 proteins was primarily related to endocytosis and cholesterol metabolism (Fig. 5B).

In addition, the top 100 *ACBD3*-related target genes were retrieved, and a Venn diagram identified the four genes at the intersection of the *ACBD3*-binding and *ACBD3*-related target genes as: *ARF1*, *BLZF1*, *BPNT1*, and *GORASP2* (Fig. 5C). The expression levels of these four genes were closely related to those of *ACBD3*: *ARF1* (*R* = 0.65), *BLZF1* (*R* = 0.70), *BPNT1* (*R* = 0.67), and *GORASP2* (*R* = 0.62) (Fig. 5D–G).

### Diagnostic value of *ACBD3* in pan-cancer

**Fig. 6:**
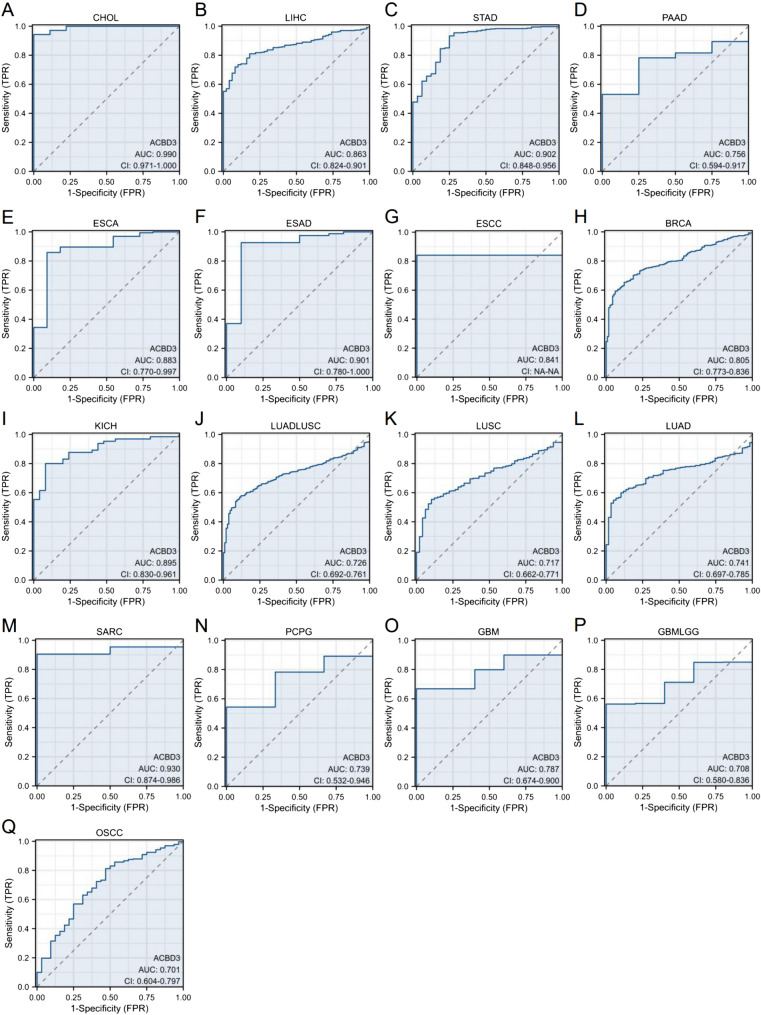
Correlations between *ACBD3* expression and receiver operating characteristic (ROC) curve across TCGA tumors, area under the ROC curve (AUC) value > 0.7 was considered to have a diagnostic value. As a measure of diagnostic accuracy, the value of AUC is closer to 1, the diagnostic value is higher. **A** cholangiocarcinoma (CHOL) (AUC = 0.990), **B** liver hepatocellular carcinoma (LIHC) (AUC = 0.863), **C** stomach adenocarcinoma (STAD) (AUC = 0.902), **D** pancreatic adenocarcinoma (PAAD) (AUC = 0.756), **E** esophageal carcinoma (ESCA) (AUC = 0.883), **F** esophagus adenocarcinoma (ESAD) (AUC = 0.901), **G** esophagus squamous cell carcinoma (ESCC) (AUC = 0.841), **H** breast invasive carcinoma (BRCA) (AUC = 0.805), **I** kidney chromophobe (KICH) (AUC = 0.895), **J** Lung cancer (LUADLUSC) (AUC = 0.726), **K** lung squamous cell carcinoma (LUSC) (AUC = 0.717), **L** lung adenocarcinoma (LUAD) (AUC = 0.741), **M** sarcoma (SARC) (AUC = 0.930), **N** pheochromocytoma and paraganglioma (PCPG) (AUC = 0.739), **O** glioblastoma multiforme (GBM) (AUC = 0.787), **P** Glioma (GBMLGG) (AUC = 0.708), **Q** Oral squamous cell carcinoma (OSCC) (AUC = 0.701)

The ROC curve was drawn to explore the diagnostic value of *ACBD3* in different cancers, and an AUC value > 0.7 was considered to have a diagnostic value. As shown, *ACBD3* had an accurate diagnostic value for 17 kinds of cancers, including CHOL (AUC = 0.990), LIHC (AUC = 0.863), STAD (AUC = 0.902), PAAD (AUC = 0.756), ESCA (AUC = 0.883), esophagus adenocarcinoma (ESAD) (AUC = 0.901), esophagus squamous cell carcinoma (ESCC) (AUC = 0.841), BRCA (AUC = 0.805), KICH (AUC = 0.895), Lung cancer (LUADLUSC) (AUC = 0.726), LUSC (AUC = 0.717), LUAD (AUC = 0.741), SARC (AUC = 0.930), PCPG (AUC = 0.739), GBM (AUC = 0.787), Glioma (GBMLGG) (AUC = 0.708), and Oral squamous cell carcinoma (OSCC) (AUC = 0.701) (Fig. 6).

### Prognostic value of *ACBD3* in pan-cancer

**Fig. 7:**
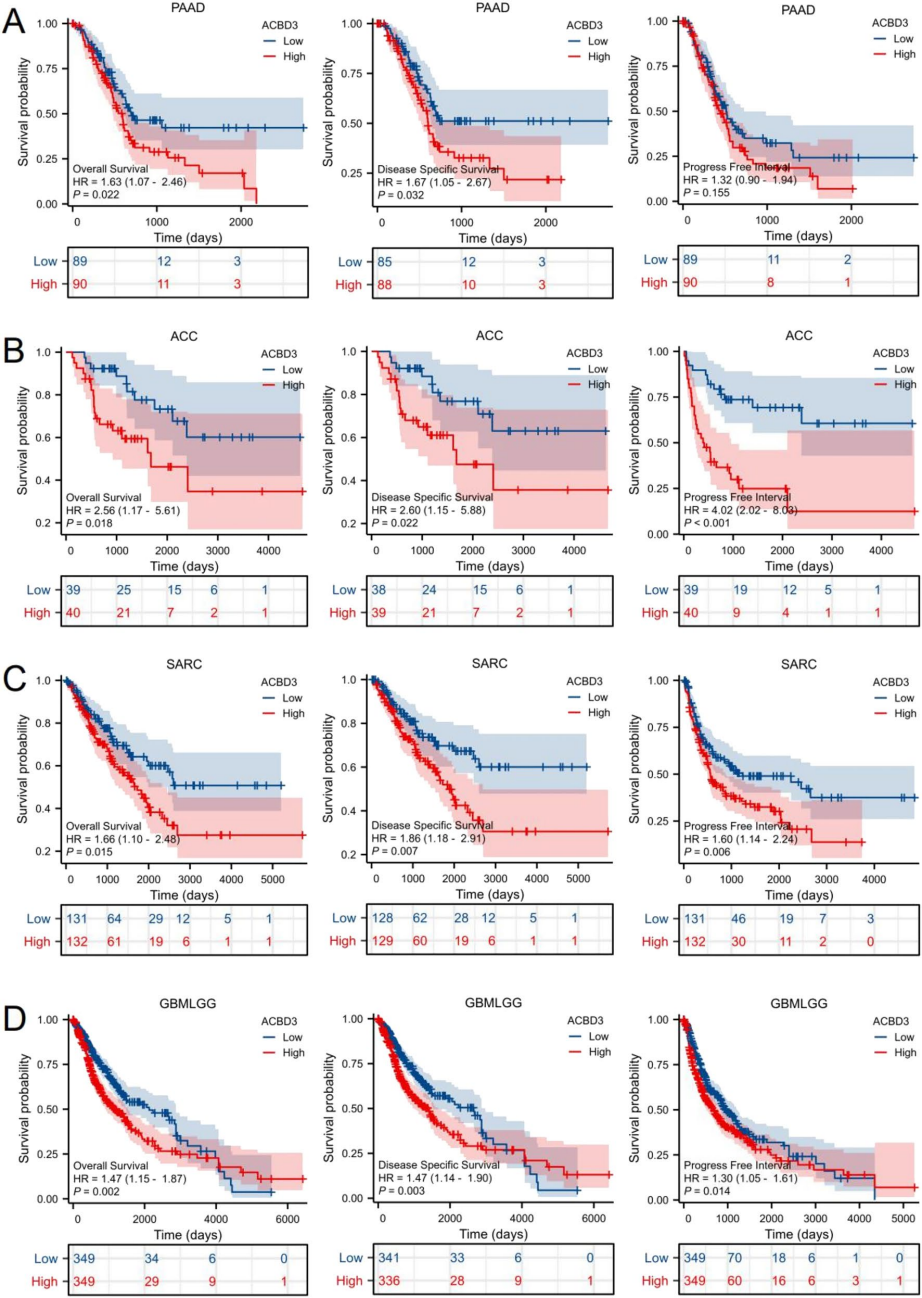
Kaplan–Meier plots [Overall Survival (OS), Disease Specific Survival (DSS), and Progress Free Interval (PFI)]for *ACBD3* expression in pan-cancers. **A** For pancreatic adenocarcinoma (PAAD), the prognosis was negatively related to *ACBD3* expression, including OS [hazard ratio (HR) = 1.63, 95% confidence interval (CI): 1.07–2.46, *p* = 0.022], DSS (HR = 1.67, 95% CI: 1.05–2.67, *p* = 0.032), and PFI (HR = 1.32, 95% CI: 0.90 -1.94, *p* = 0.155). **B** For adrenocortical carcinoma (ACC), the prognosis was negatively related to *ACBD3* expression, including OS (HR = 2.56, 95% CI: 1.17–5.61, *p* = 0.018), DSS (HR = 2.60, 95% CI: 1.15–5.88, *p* = 0.022), and PFI (HR = 4.02, 95% CI: 2.02–8.03, *p* < 0.001). **C** For sarcoma (SARC), the prognosis was negatively related to *ACBD3* expression, including OS (HR = 1.66, 95% CI: 1.10–2.48, *p* = 0.015), DSS (HR = 1.86, 95% CI: 1.18–2.91, *p* = 0.007), and PFI (HR = 1.60, 95% CI: 1.14–2.24, *p* = 0.006). **D** For glioma (GBMLGG), the prognosis was negatively related to *ACBD3* expression, including OS (HR = 1.47, 95% CI: 1.15–1.87, *p* = 0.002), DSS (HR = 1.47, 95% CI: 1.14–1.90, *p* = 0.003), and PFI (HR = 1.30, 95% CI: 1.05–1.61, *p* = 0.014

Figure 7 showed that the OS, DSS, and PFI of four tumors were closely correlated with the expression levels of *ACBD3*, including PAAD, adrenocortical carcinoma (ACC), SARC, and GBMLGG. As for PAAD, the prognosis was negatively related to *ACBD3* expression, including OS [hazard ratio (HR) = 1.63, 95% confidence interval (CI): 1.07–2.46, *p* = 0.022], DSS (HR = 1.67, 95% CI: 1.05–2.67, *p* = 0.032), and PFI (HR = 1.32, 95% CI: 0.90–1.94, *p* = 0.155). As for ACC, the prognosis was negatively related to *ACBD3* expression, including OS (HR = 2.56, 95% CI: 1.17–5.61, *p* = 0.018), DSS (HR = 2.60, 95% CI: 1.15–5.88, *p* = 0.022), and PFI (HR = 4.02, 95% CI: 2.02–8.03, *p* < 0.001). Also, for SARC, the prognosis was negatively related to *ACBD3* expression, including OS (HR = 1.66, 95% CI: 1.10–2.48, *p* = 0.015), DSS (HR = 1.86, 95% CI: 1.18–2.91, *p* = 0.007), and PFI (HR = 1.60, 95% CI: 1.14–2.24, *p* = 0.006). And for GBMLGG, the prognosis was negatively related to *ACBD3* expression, including OS (HR = 1.47, 95% CI: 1.15–1.87, *p* = 0.002), DSS (HR = 1.47, 95% CI: 1.14–1.90, *p* = 0.003), and PFI (HR = 1.30, 95% CI: 1.05–1.61, *p* = 0.014).

We used the GSE57495, GSE83300, and GSE19750 in the GEO database for validation and found that the OS prognosis was negatively related to *ACBD3* expression in GBMLGG (HR = 2.30, 95% CI: 1.19–4.43, *p* = 0.013). However, there was no statistically significant relationship between *ACBD3* expression and prognosis in PAAD and ACC (Additional file 1: Fig. S3).

### Genetic alteration and DNA methylation analysis of *ACBD3* in pan-cancers

**Fig. 8:**
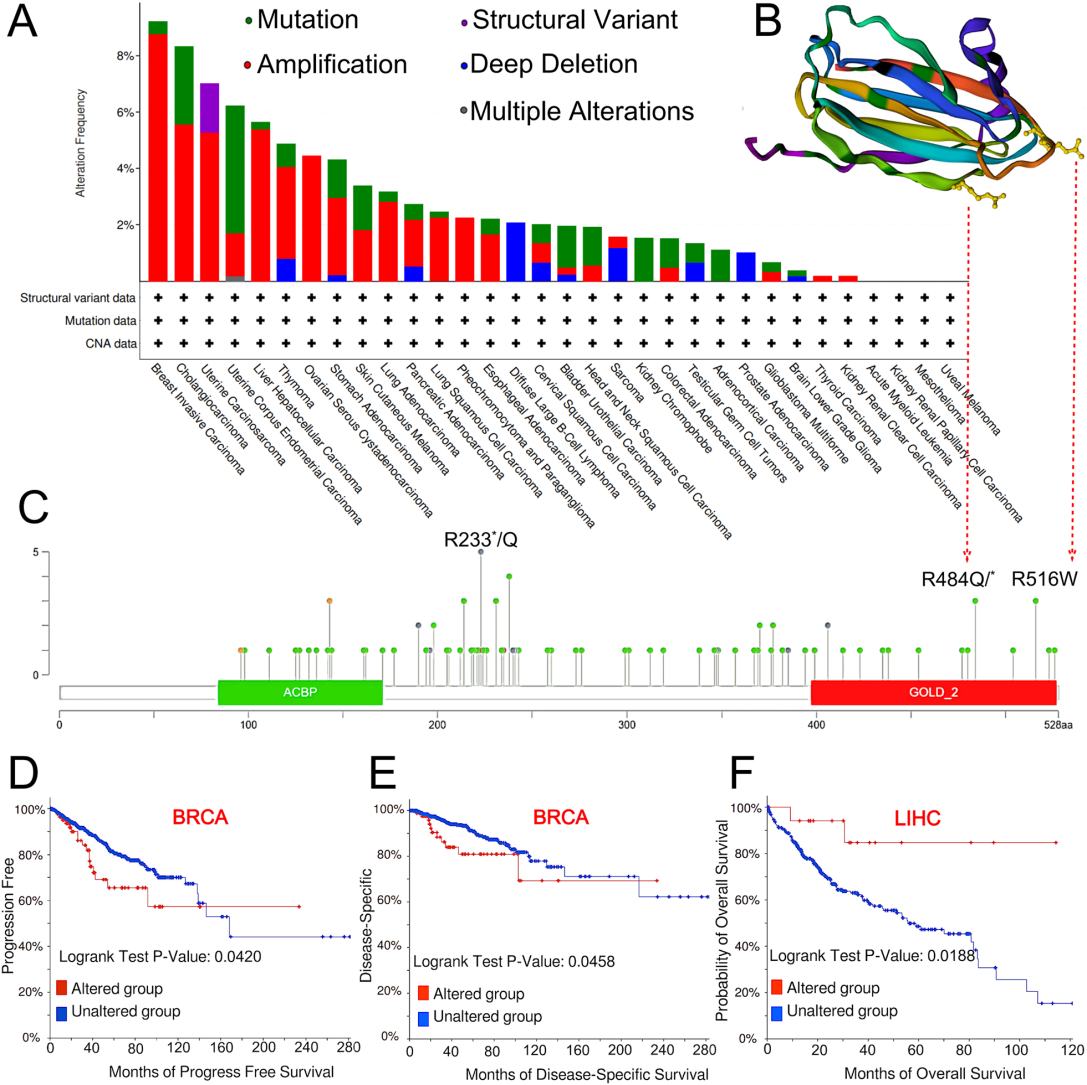
*ACBD3* mutation in various tumors of TCGA. **A** The alteration type and frequency. Among the many alteration types of *ACBD3*, “Amplification” was the most common type in patients with breast invasive carcinoma (BRCA) (9.22% alteration frequency) and liver hepatocellular carcinoma (LIHC) (5.38% alteration frequency). **B** The 3D structure of ACBD3 protein. **C** The number, sites, and types of the *ACBD3* genetic alteration. “Missense Mutation” was the most dominant type of mutation. The alteration of R484Q/* and R516W in GOLD-2 is able to lead to the missense mutation of *ACBD3*. **D**–**F** The correlation between *ACBD3* alternation and clinical outcomes in different tumors. For BRCA, cases with *ACBD3* alternation had a worse prognosis in both progression-free survival and disease-specific survival. Patients with LIHC with *ACBD3* alternation had a better prognosis in overall survival

The gene alteration characteristics of *ACBD3* in TCGA pan-cancer atlas were obtained. We found that among the many alteration types of *ACBD3*, “Amplification” was the most common type in patients with BRCA (9.22% alteration frequency) and LIHC (5.38% alteration frequency) (Fig. 8A). It is worth noting that, “Amplification” was the only type of genetic alteration in ovarian serous cystadenocarcinoma, pheochromocytoma, and paraganglioma (Fig. 8A). “Mutation” is the only type of genetic alteration in kidney chromophobe and adrenocortical carcinoma cases (Fig. 8A). “Deep Deletion” is the only type of genetic alteration in diffuse large B-cell lymphoma and prostate adenocarcinoma (Fig. 8A). Figure 8B displayed the 3D structure of *ACBD3* protein. Figure 8C displayed the number, sites, and types of the *ACBD3* genetic alteration. As shown in Fig. 8C, “Missense Mutation” was the most dominant type of mutation. Alteration in R484Q/ * and R516W in GOLD-2 could have led to the missense mutations in *ACBD3*.

In addition, we determined the relationship between *ACBD3* alterations and clinical outcomes in BRCA and LIHC. For BRCA, patients with *ACBD3* alterations had a worse prognosis in terms of progression-free survival (Fig. 8D) and disease-specific survival (Fig. 8E) than those patients without *ACBD3* alterations. Patients with LIHC and *ACBD3* alteration had a better prognosis in overall survival than those patients without *ACBD3* alterations (Fig. 8F).

**Fig. 9:**
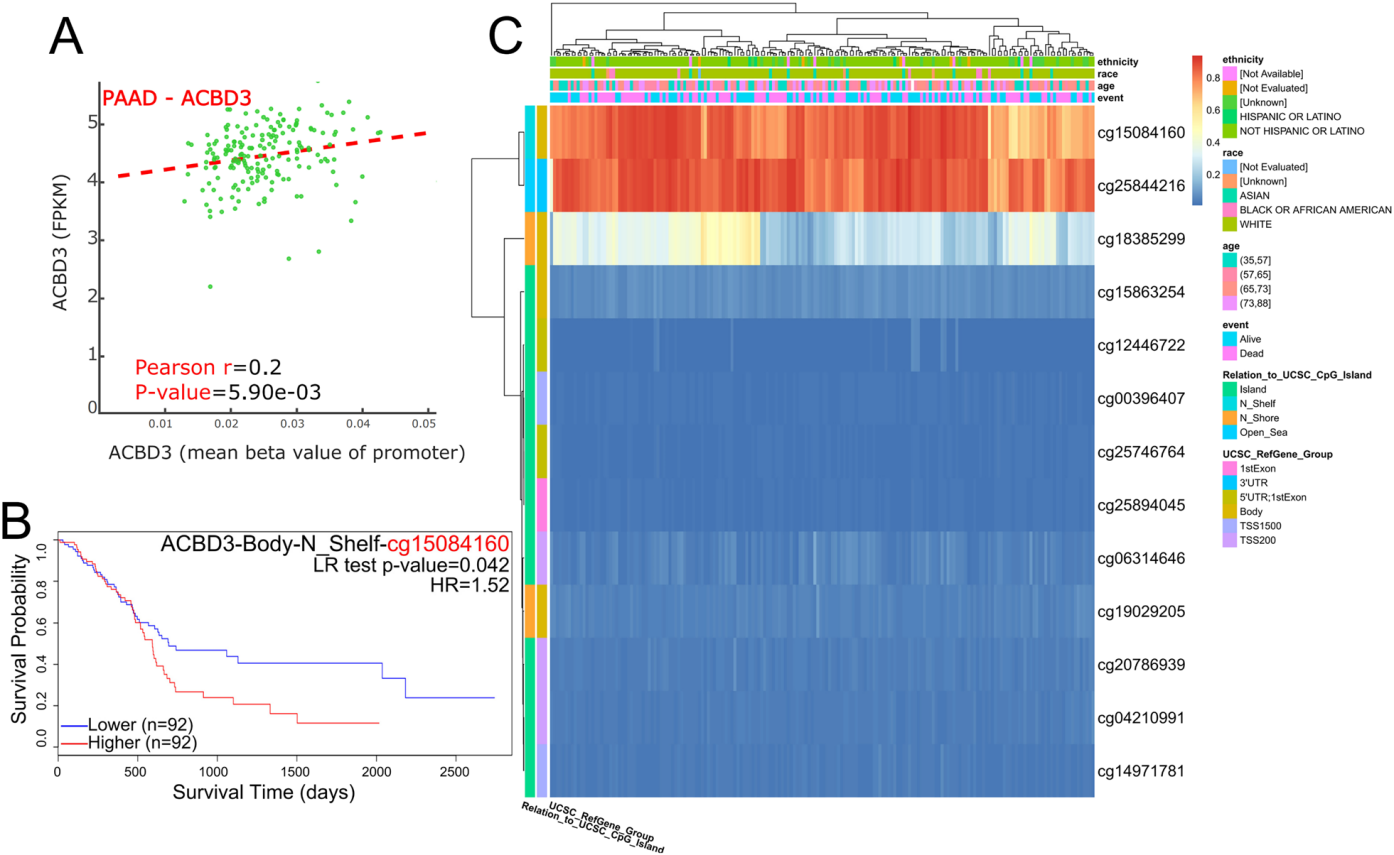
The correlation between the methylation level and *ACBD3* expression in pancreatic adenocarcinoma (PAAD). **A** *ACBD3* expression was positive related with *ACBD3* promoter methylation in PAAD (Pearson *r* = 0.2). **B** Kaplan–Meier plots for *ACBD3* promoter methylation in PAAD. the higher methylation β values of *ACBD3*-Body-N_Shelf-cg15084160 lead to a worse Overall Survival prognosis in PAAD. **C** The heat map of the relationship between *ACBD3* expression and the methylation level in PAAD

Furthermore, we accessed the relationship between DNA methylation and *ACBD3* expression and found that promoter methylation was positively correlated with *ACBD3* expression in PAAD (Pearson r = 0.2) (Fig. 9A). In addition, higher methylation β values of *ACBD3*-Body-N_Shelf-cg15084160 led to a worse OS prognosis in PAAD [HR = 1.52, CI(1.014;2.281), P < 0.05] (Fig. 9B and Table 1).

**Table 1:** Association of ACBD3 with methylation sites in pan-cancers

CpG site	Cancer	Gene	Group	CpG Island	HR	CI	*P*
cg20786939	KIRC	ACBD3	TSS200	Island	0.362	(0.208;0.632)	< 0.001
cg18385299	LGG	ACBD3	Body	N_Shore	0.382	(0.264;0.551)	< 0.001
cg25844216	LGG	ACBD3	3'UTR	Open_Sea	0.573	(0.394;0.834)	0.004
cg15084160	PAAD	ACBD3	Body	N_Shelf	1.52	(1.014;2.281)	0.043

*HR* hazard ratio, *CI* confidence interval, *KIRC* kidney renal clear cell carcinoma, *LGG* brain lower grade glioma, *PAAD* pancreatic adenocarcinoma

### Protein phosphorylation analysis

**Fig. 10:**
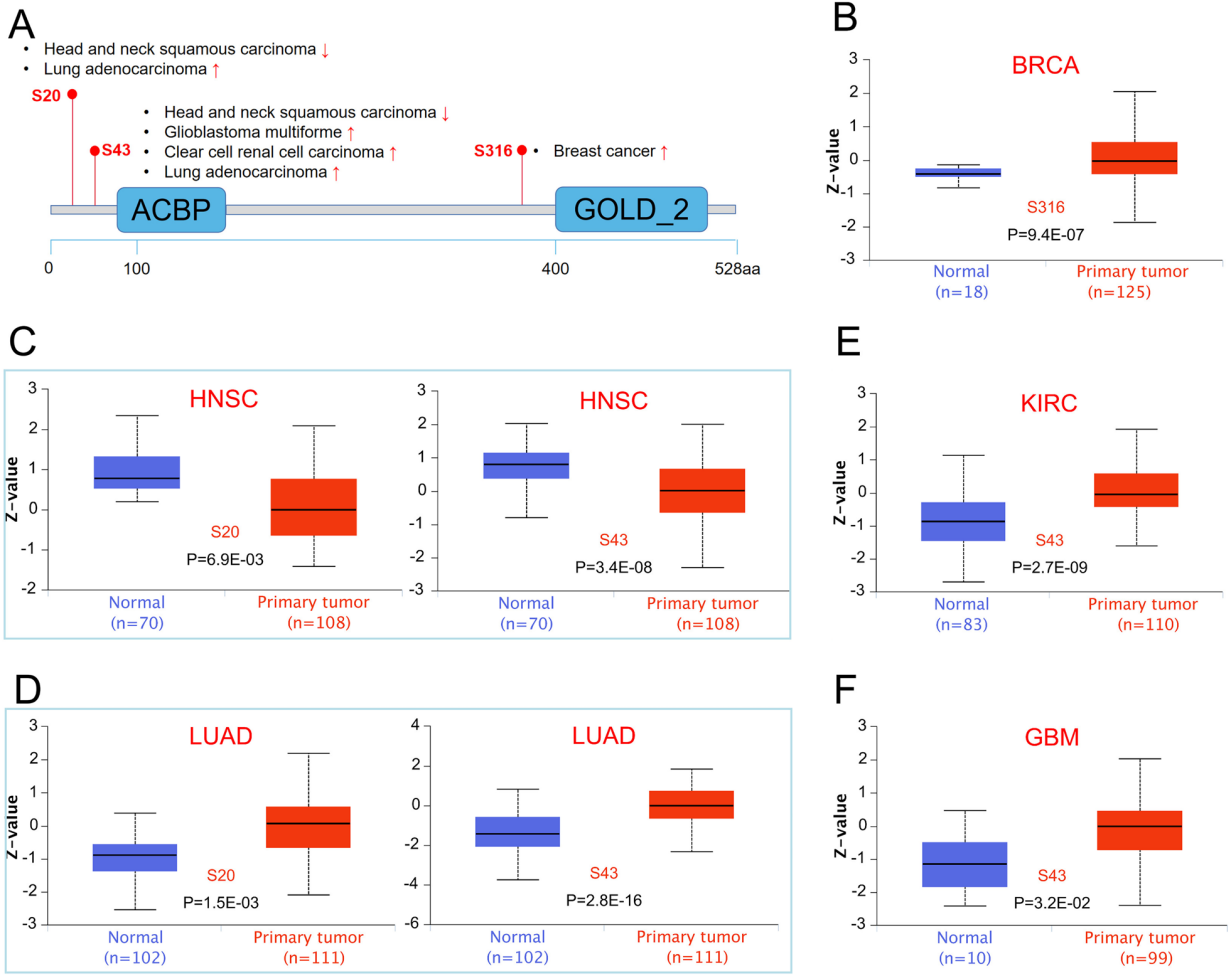
Phosphorylation levels of *ACBD3* protein in various tumors. **A** Summary of the correlation between various cancers and *ACBD3* phosphorylation sites; **B** breast invasive carcinoma (BRCA); **C** head and neck squamous cell carcinoma (HNSC); **D** lung adenocarcinoma (LUAD); **E** kidney renal clear cell carcinoma (KIRC); **F** glioblastoma multiforme (GBM). The S316 locus expressed an upper phosphorylation level in BRCA when compared with normal tissues. The S20 locus exhibited a lower phosphorylation level in HNSC, and LUAD is the opposite. The S43 locus expressed an upper phosphorylation level in KIRC, LUAD, and GBM, and decreased in HNSC

Figure 10A summarized the correlation between various cancers and *ACBD3* phosphorylation sites. The S316 locus showed an increased phosphorylation level in BRCA when compared with normal tissues (Fig. 10B). Compared with normal tissues, the S20 locus exhibited a lower phosphorylation level in HNSC (Fig. 10C), with LUAD (Fig. 10D) exhibited the opposite trend. The phosphorylation levels at S43 were higher in KIRC (Fig. 10E), LUAD (Fig. 10D), and GBM (Fig. 10F) and decreased in HNSC (Fig. 10C).

## Discussion

Known as *GCP60*, *ACBD3* majored in maintaining the structure and function of the Golgi apparatus. Changes in Golgi structure and function are closely related to cancer development, and Golgi-associated proteins may help diagnose cancer and guide treatment [24–26]. Since the Golgi apparatus is mainly involved in the synthesis and redistribution of new proteins, we speculate that *ACBD3* promotes protein binding and thus plays significant roles in the occurrence and development of many tumors with different characteristics. Previous studies demonstrated that *ACBD3* was involved in the development and treatment of various types of cancers [11–13]. Nevertheless, no studies have explored the function of *ACBD3* in pan-cancers systematically. In order to gain a more comprehensive understanding of *ACBD3*, we are the first to explore its function and expression of *ACBD3* in pan-cancers from the perspective of bioinformatic analysis.

By exploring the TCGA database, we found that *ACBD3* expression was remarkably upregulated in eleven cancers, and downregulated in three cancers. This finding suggests that *ACBD3* regulates the formation and replication of tumors and facilitates the development of most cancers by acting as an oncogenic gene. We investigated the correlation between *ACBD3* expression and the molecular and immune subtypes of TCGA tumors and found that the molecular and immune subtypes of HNSC, STAD, SKCM, OV, LUSC, and LIHC were related to *ACBD3* expression. We have assumed that *ACBD3* might play a potential role in the occurrence of tumor subtypes, and more experimental results are needed to support this theory. In addition, analysis of the molecular and immune subtypes of various malignant tumors provides a research direction for new tumor therapeutic targets.

We identified ten proteins that interacted most closely with *ACBD3*: *GOLGA3*, *PI4KB*, *ARF1*, *GOLPH3*, *OSBP*, *TGOLN2*, *GORASP2*, *GOLGB1*, *GORASP1*, and *GBF1*. The GO|KEGG pathway enrichment analysis suggested that “Golgi organization” and “protein kinase A binding” is the main function of *ACBD3*, which confirms our hypothesis about the function of the *ACBD3*-binding proteins. Previous studies had revealed that protein kinase A (PKA) is involved in cancer transformation [27]. The occurrence and development of LIHC, OV, GBM, and ESCC are closely related to PKA [28–31], which reflects the potential association between *ACBD3* and various tumors.

What’s more, exploration of the relationship between *ACBD3* expression and the diagnostic value of various malignant tumors with different biological characteristics showed that *ACBD3* can be used to diagnose a variety of cancers, including CHOL, LIHC, STAD, PAAD, ESCA, ESAD, ESCC, BRCA, KICH, LUADLUSC, LUSC, LUAD, SARC, PCPG, GBM, GBMLGG, and OSCC. Notably, *ACBD3* had high diagnostic value (AUC > 0.9) for CHOL, STAD, ESAD, and SARC. In addition, the K-M survival curve for various cancers revealed that *ACBD3* was closely associated with the prognosis in PAAD, ACC, SARC, and GBMLGG. Because of the difficulty of integrating all sarcoma-related data, we verified the remaining three results using the GEO database and found that only the prognosis of GBMLGG was correlated with *ACBD3* expression. We cannot rule out that this negative result is due to the small amount of data in the GEO database. Furthermore, we found that higher methylation β values of *ACBD3*-Body-N_Shelf-cg15084160 led to worse OS prognosis in PAAD. These discoveries indicate that *ACBD3* has a very important diagnostic and prognostic significance in most cancers, and is expected to be a new biomarker for pan-carcinoma.

In addition, *ACBD3* gene mutation analysis has shown that *ACBD3* mutations exist in a variety of tumor cells, with missense mutations being the most common. The missense mutations of R484Q/ * and R516W in GOLD-2 can lead to missense mutations in *ACBD3*. In BRCA, the *ACBD3* altered group had poor prognosis, whereas the reverse was true for LIHC. These results provide a new direction for evaluating the prognosis.

Phosphorylation is one of the most extensive post-translational modifications and plays an important role in regulating cell growth, differentiation, apoptosis, and cell signaling [32]. Kinase inhibitors are also considered valuable for the treatment of tumors [33]. Thus, we investigated the phosphorylation levels of *ACBD3* in BRCA, HNSC, KIRC, LUAD, and GBM. We found that the phosphorylation levels of *ACBD3* at various phosphorylation sites decreased in HNSC, but increased in BRCA, KIRC, LUAD, and GBM. This discovery could lead to further research on the molecular mechanisms and potential therapeutic targets for tumors. However, we cannot get rid of the possibility that the difference in phosphorylation levels is a by-product of meaningless signal dysregulation. Therefore, further experimental verification is required.

Previous research had revealed that CAF was involved in various cancers developing [34]. We discovered that *ACBD3* expression positively related to CAF infiltration in HNSC. Besides, *ACBD3* expression was also different in various immune subtypes of HNSC, which may indicate a correlation between the occurrence and development of HNSC and the infiltration of CAF.

The advantage of this study is that we reflected the expression and clinical value of *ACBD3* in pan-cancers using a variety of databases in a comprehensive and systematic manner. Secondly, this was the first study to analyze the biological significance of *ACBD3* in pan-cancers and obtain relatively comprehensive results.

However, our study has several limitations. First of all, we only used the existing RNA-seq and clinical data of cancers in online databases for analysis but lacked actual clinical data. Secondly, there is need to conduct further biological experiments to verify our conclusions. Currently, various bioinformatic analysis methods are available. In future studies, we plan to combine various learning methods, such as machine learning, to further understand the function of *ACBD3* in various cancers.

In summary, we found a statistically significant relationship between *ACBD3* expression and immune subtypes, molecular subtypes, diagnosis, prognosis, tumor mutation burden, protein phosphorylation levels, and immune cell infiltration in pan-cancers. This comprehensive and systematic pan-cancer analysis of *ACBD3* supports further explorations into the critical role of *ACBD3* during the development of tumors and offer a comprehensive analytical basis for further molecular, biological, and experimental verification in future clinical decisions.

## Conclusion

According to our pan-cancer analysis of *ACBD3*, *ACBD3* may serve as a novel prognostic and diagnostic biomarker for pan-cancers as it contributes to tumor development. As such, *ACBD3* may also provide new directions for cancer treatment targets in the future.

### Supplementary Information

**Additional file 1: Figure S1** The expression level of ACBD3. **A** In normal tissues, ACBD3 expressed highly in cerebral cortex, hippocampus, duodenum, small intestine, colon, gallbladder, pancreas, prostate, placenta, appendix, and bone marrow. Moreover, ACBD3 expressed lowly in oral mucosa, liver, ovary, soft tissue, and adipose tissue; **B** In tumor cell lines, The expression of ACBD3 ranks high in breast cancer, kidney cancer, and myeloma in tumor cell lines; **C**, **D** Intracellular ACBD3 is mainly distributed in Golgi; **E** ACBD3 expressed high in SKCM, BRCA, GBM, KIRC, LGG, and THCA in Cancer Cell Line Encyclopedia (CCLE) database. Small cell lung cancer (SCLC), colon and rectal cancer (COADREAD), large b-cell lymphoma (DLBC), sarcoma (SARC), multiple myeloma (MM), acute myeloid leukemia (LAML), skin cutaneous melanoma (SKCM), breast cancer (BRCA), liver hepatocellular carcinoma (LIHC), mesothelioma (MESO), ovarian cancer (OV), esophageal cancer (ESCA), endometrioid cancer (UCEC), glioblastoma multiforme (GBM), pancreatic adenocarcinoma (PAAD), neuroblastoma (NB), lung adenocarcinoma (LUAD), stomach adenocarcinoma (STAD), kidney clear cell carcinoma (KIRC), non-small cell lung carcinoma (NSC), acute lymphoeytic leukemia (ALL), lung squamous cell carcinoma (LUSC), brain lower grade glioma (LGG), head and neck cancer (HNSC), thyroid cancer (THCA), chronic myelocytic leukemia (LCML), Human medulloblastoma cells (MB), prostate cancer (PRAD), cervical cancer (CESC), chronic lymphocytic leukemia (CLL). **Figure S2** Correlation between immune infiltrates and *ACBD3* expression in different tumors. head and neck cancer (HNSC), bladder cancer (BLCA), colon cancer (COAD), thyroid cancer (THCA), kidney clear cell carcinoma (KIRC). **A** The expression of *ACBD3* was actively correlated with the cancer-associated fibroblast (CAF) infiltration for HNSC. **B** The expression of *ACBD3* was positively related to neutrophil infiltration for BLCA, COAD, and THCA. **C** *ACBD3* expression was positively related to endothelial cell infiltration for COAD, HNSC, and KIRC. **Figure S3** Kaplan–Meier plots ([Overall Survival (OS)]) for *ACBD3* expression in pan-cancers. **A** Pancreatic For pancreatic adenocarcinoma (PAAD), there was no statistically significant relationship between *ACBD3* expression and prognosis. **B** For Glioma (GBMLGG), the OS prognosis was negatively related to *ACBD3* expression, HR = 2.30, 95% CI: 1.19–4.43, *p* = 0.013. **C** For adrenocortical carcinoma (ACC), there was no statistically significant relationship between *ACBD3* expression and prognosis.

## Data Availability

The datasets used and/or analysed during the current study are available from the corresponding author on reasonable request.
