# Supplementary material for: Identification of ACBD3 as a new molecular biomarker in pan-cancers through bioinformatic analysis: a preclinical study
Source: Eur J Med Res. 2023 Dec 14;28:590. doi: 10.1186/s40001-023-01576-8 (PMC10720239; doi:10.1186/s40001-023-01576-8)
Supplement: Supplementary file 1 — Additional file 1: Figure S1 The expression level of ACBD3. A In normal tissues, ACBD3 expressed highly in cerebral cortex, hippocampus, duodenum, small intestine, colon, gallbladder, pancreas, prostate, placenta, appendix, and bone marrow. Moreover, ACBD3 expressed lowly in oral mucosa, liver, ovary, soft tissue, and adipose tissue; B In tumor cell lines, The expression of ACBD3 ranks high in breast cancer, kidney cancer, and myeloma in tumor cell lines; C, D Intracellular ACBD3 is mainly distributed in Golgi; E ACBD3 expressed high in SKCM, BRCA, GBM, KIRC, LGG, and THCA in Cancer Cell Line Encyclopedia (CCLE) database. Small cell lung cancer (SCLC), colon and rectal cancer (COADREAD), large b-cell lymphoma (DLBC), sarcoma (SARC), multiple myeloma (MM), acute myeloid leukemia (LAML), skin cutaneous melanoma (SKCM), breast cancer (BRCA), liver hepatocellular carcinoma (LIHC), mesothelioma (MESO), ovarian cancer (OV), esophageal cancer (ESCA), endometrioid cancer (UCEC), glioblastoma multiforme (GBM), pancreatic adenocarcinoma (PAAD), neuroblastoma (NB), lung adenocarcinoma (LUAD), stomach adenocarcinoma (STAD), kidney clear cell carcinoma (KIRC), non-small cell lung carcinoma (NSC), acute lymphoeytic leukemia (ALL), lung squamous cell carcinoma (LUSC), brain lower grade glioma (LGG), head and neck cancer (HNSC), thyroid cancer (THCA), chronic myelocytic leukemia (LCML), Human medulloblastoma cells (MB), prostate cancer (PRAD), cervical cancer (CESC), chronic lymphocytic leukemia (CLL). Figure S2 Correlation between immune infiltrates and ACBD3 expression in different tumors. head and neck cancer (HNSC), bladder cancer (BLCA), colon cancer (COAD), thyroid cancer (THCA), kidney clear cell carcinoma (KIRC). A The expression of ACBD3 was actively correlated with the cancer-associated fibroblast (CAF) infiltration for HNSC. B The expression of ACBD3 was positively related to neutrophil infiltration for BLCA, COAD, and THCA. C ACBD3 expression was positively related to e [file 40001_2023_1576_MOESM1_ESM.docx]

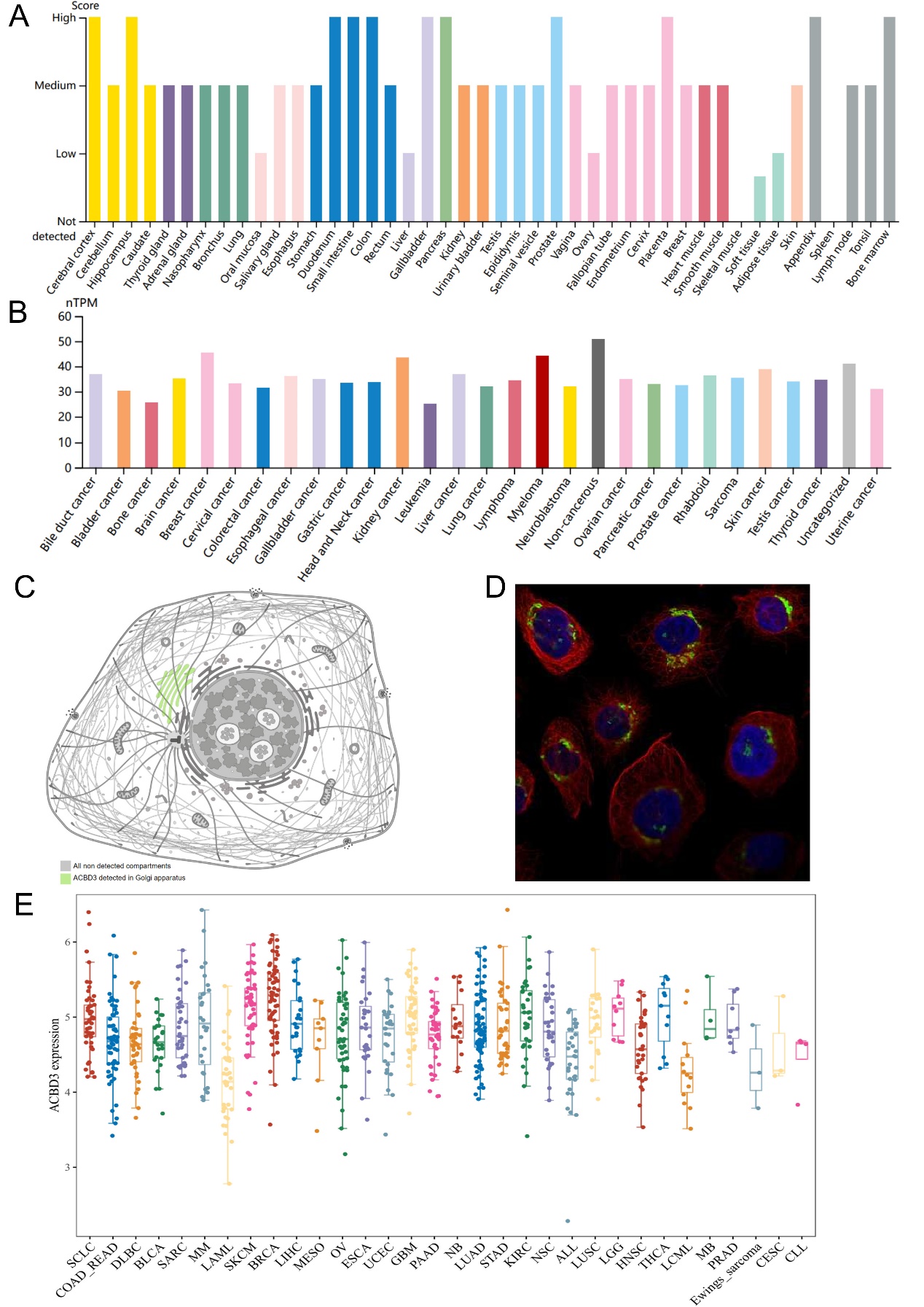


**Additional file 1: Figure S1** The expression level of ACBD3. (A) In normal tissues, ACBD3 expressed highly in cerebral cortex, hippocampus, duodenum, small intestine, colon, gallbladder, pancreas, prostate, placenta, appendix, and bone marrow. Moreover, ACBD3 expressed lowly in oral mucosa, liver, ovary, soft tissue, and adipose tissue; (B) In tumor cell lines, The expression of ACBD3 ranks high in breast cancer, kidney cancer, and myeloma in tumor cell lines; (C-D) Intracellular ACBD3 is mainly distributed in Golgi; (E) ACBD3 expressed high in SKCM, BRCA, GBM, KIRC, LGG, and THCA in Cancer Cell Line Encyclopedia (CCLE) database. Small cell lung cancer (SCLC), colon and rectal cancer (COADREAD), large b-cell lymphoma (DLBC), sarcoma (SARC), multiple myeloma (MM), acute myeloid leukemia (LAML), skin cutaneous melanoma (SKCM), breast cancer (BRCA), liver hepatocellular carcinoma (LIHC), mesothelioma (MESO), ovarian cancer (OV), esophageal cancer (ESCA), endometrioid cancer (UCEC), glioblastoma multiforme (GBM), pancreatic adenocarcinoma (PAAD), neuroblastoma (NB), lung adenocarcinoma (LUAD), stomach adenocarcinoma (STAD), kidney clear cell carcinoma (KIRC), non-small cell lung carcinoma (NSC), acute lymphoeytic leukemia (ALL), lung squamous cell carcinoma (LUSC), brain lower grade glioma (LGG), head and neck cancer (HNSC), thyroid cancer (THCA), chronic myelocytic leukemia (LCML), Human medulloblastoma cells (MB), prostate cancer (PRAD), cervical cancer (CESC), chronic lymphocytic leukemia (CLL).


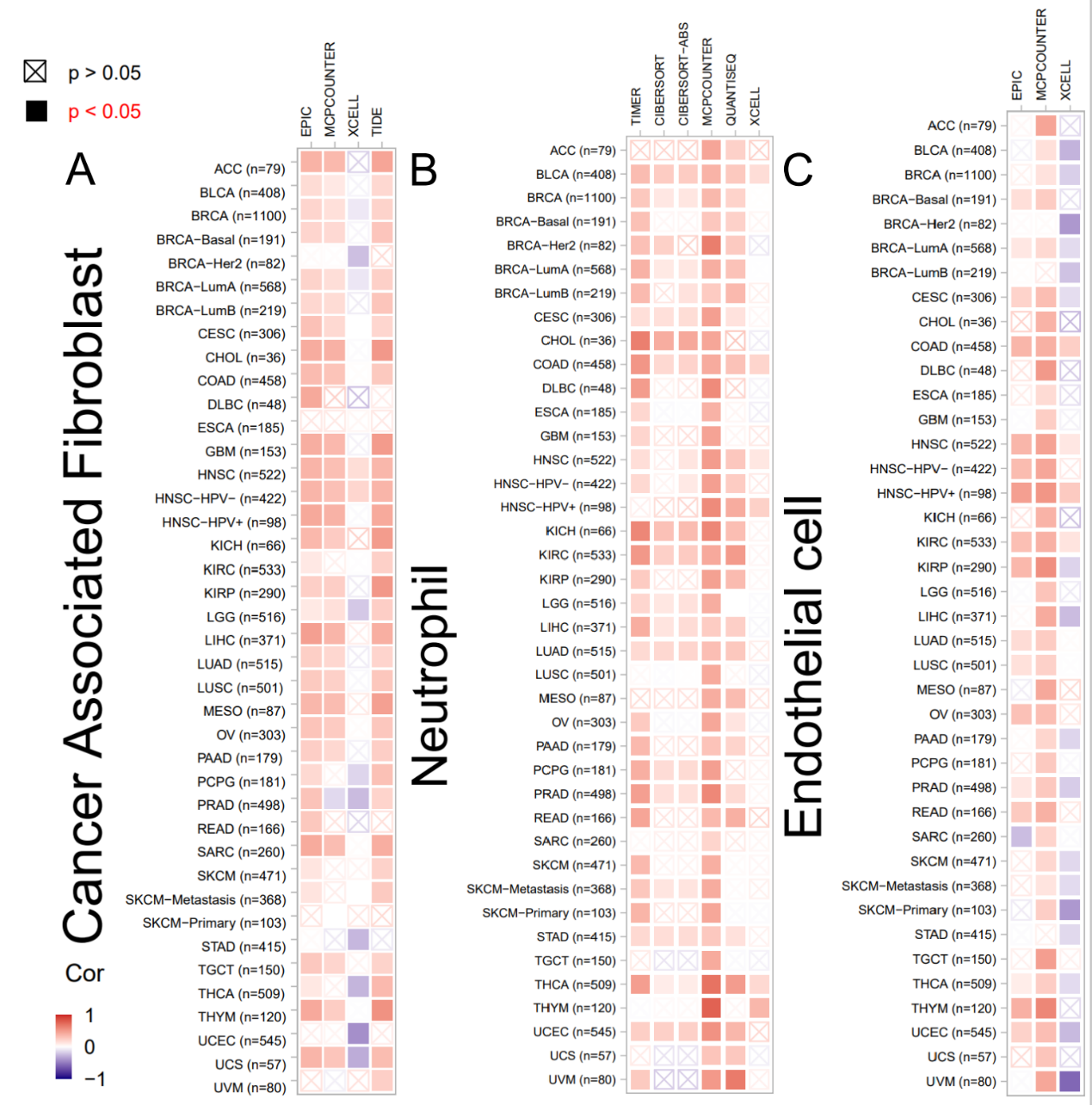


**Additional file 1: Figure S2** Correlation between immune infiltrates and *ACBD3* expression in different tumors. head and neck cancer (HNSC), bladder cancer (BLCA), colon cancer (COAD), thyroid cancer (THCA), kidney clear cell carcinoma (KIRC). (A) The expression of *ACBD3* was actively correlated with the cancer-associated fibroblast (CAF) infiltration for HNSC. (B) The expression of *ACBD3* was positively related to neutrophil infiltration for BLCA, COAD, and THCA. (C) *ACBD3* expression was positively related to endothelial cell infiltration for COAD, HNSC, and KIRC.


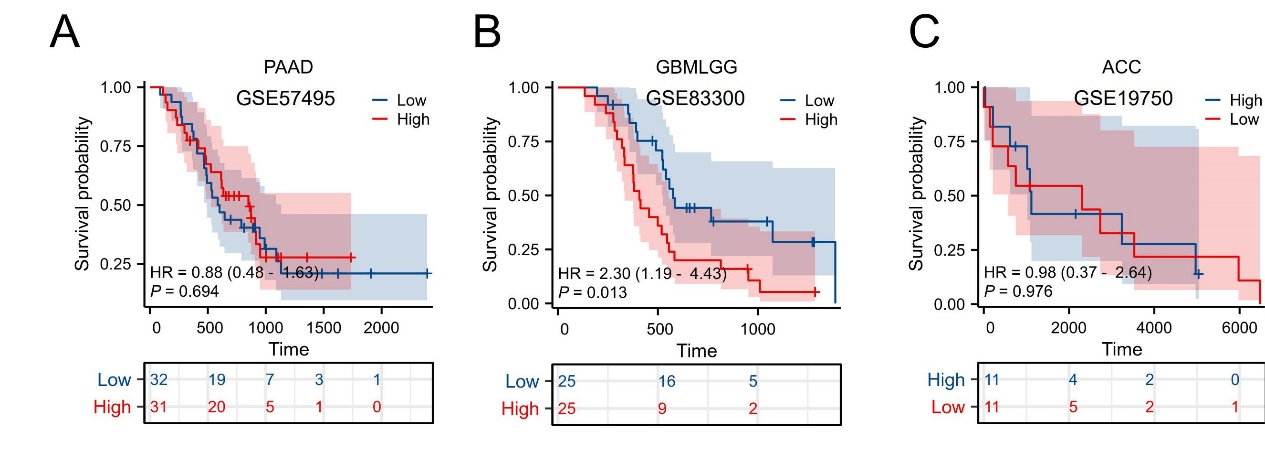


**Additional file 1: Figure 3** Kaplan–Meier plots ([Overall Survival (OS)] for *ACBD3* expression in pan-cancers. (A) Pancreatic For pancreatic adenocarcinoma (PAAD), there was no statistically significant relationship between *ACBD3* expression and prognosis. (B) For Glioma (GBMLGG), the OS prognosis was negatively related to *ACBD3* expression, HR = 2.30, 95% CI: 1.19 – 4.43, p = 0.013. (C) For adrenocortical carcinoma (ACC), there was no statistically significant relationship between *ACBD3* expression and prognosis.
